# Crossmodal Semantic Congruence Interacts with Object Contextual Consistency in Complex Visual Scenes to Enhance Short-Term Memory Performance

**DOI:** 10.3390/brainsci11091206

**Published:** 2021-09-13

**Authors:** Erika Almadori, Serena Mastroberardino, Fabiano Botta, Riccardo Brunetti, Juan Lupiáñez, Charles Spence, Valerio Santangelo

**Affiliations:** 1 Neuroimaging Laboratory, IRCCS Santa Lucia Foundation, Via Ardeatina 306, 00179 Rome, Italy; e.almadori@gmail.com; 2 Department of Psychology, School of Medicine & Psychology, Sapienza University of Rome, Via dei Marsi 78, 00185 Rome, Italy; serena.mastroberardino@uniroma1.it; 3 Department of Experimental Psychology and Mind, Brain, and Behavior Research Center (CIMCYC), University of Granada, 18071 Granada, Spain; fabianobotta@ugr.es (F.B.); jlupiane@ugr.es (J.L.); 4 Cognitive and Clinical Psychology Laboratory, Department of Human Sciences, Università Europea di Roma, 00163 Roma, Italy; riccardo.brunetti@unier.it; 5 Department of Experimental Psychology, Oxford University, Oxford OX2 6GG, UK; Charles.Spence@psy.ox.ac.uk; 6 Department of Philosophy, Social Sciences & Education, University of Perugia, Piazza G. Ermini, 1, 06123 Perugia, Italy

**Keywords:** short-term memory, semantics, visual scenes, auditory, multisensory, spatial orienting

## Abstract

Object sounds can enhance the attentional selection and perceptual processing of semantically-related visual stimuli. However, it is currently unknown whether crossmodal semantic congruence also affects the post-perceptual stages of information processing, such as short-term memory (STM), and whether this effect is modulated by the object consistency with the background visual scene. In two experiments, participants viewed everyday visual scenes for 500 ms while listening to an object sound, which could either be semantically related to the object that served as the STM target at retrieval or not. This defined crossmodal semantically cued vs. uncued targets. The target was either in- or out-of-context with respect to the background visual scene. After a maintenance period of 2000 ms, the target was presented in isolation against a neutral background, in either the same or different spatial position as in the original scene. The participants judged the same vs. different position of the object and then provided a confidence judgment concerning the certainty of their response. The results revealed greater accuracy when judging the spatial position of targets paired with a semantically congruent object sound at encoding. This crossmodal facilitatory effect was modulated by whether the target object was in- or out-of-context with respect to the background scene, with out-of-context targets reducing the facilitatory effect of object sounds. Overall, these findings suggest that the presence of the object sound at encoding facilitated the selection and processing of the semantically related visual stimuli, but this effect depends on the semantic configuration of the visual scene.

## 1. Introduction

The multisensory environment in which we live is undoubtedly highly complex. In recent decades, a number of studies have highlighted how crossmodal interactions can affect stimulus selection and stimulus processing (see, for reviews, [1,2,3,4,5,6,7]). For instance, a stimulus presented in a given sensory modality (e.g., audition) and in a given spatial location (e.g., on the right) can affect the processing of a target stimulus presented in a different sensory modality (e.g., visual) in the same spatial location, even when the presentation of the former stimulus happens to be entirely nonpredictive with regard to the location of the target, as evidenced by research using the orthogonal cuing paradigm [8,9]. Crossmodal signals that are task irrelevant can also affect perceptual processing in nonspatial settings. For instance, a sensory feature (e.g., a high- or low-pitch tone) has been shown to aid perceptual discrimination of a stimulus in another sensory modality (e.g., a smaller or larger circle), as evidenced by the literature on crossmodal correspondences [10,11].

Task-irrelevant crossmodal stimuli can also affect selection and stimulus processing when they are semantically associated with the target stimulus. This has been evidenced using both cuing [12] and visual search paradigms [13,14,15,16,17,18]. For instance, Mastroberardino and colleagues [12] presented two pictures, one of a cat and the other of a dog, in either hemifield, synchronized with a central sound that was semantically congruent with one of the pictures (i.e., either a “meow” or a “woof” sound). This was followed by the presentation of two Gabor patches, one red and the other blue, in either hemifield. The participants had to judge the orientation of the target Gabor patch, defined by one of the colors. Participants were more accurate at discriminating the orientation of the Gabor patch when it happened to be presented in the location where the crossmodal semantically congruent stimuli had been presented (e.g., a to-be-judged Gabor on the left preceded by the picture of the cat on the left with a “meow” central sound), as compared to when the Gabor was presented on the opposite side. This result was interpreted as indicating that crossmodal semantic congruence generates an attentional and processing bias toward the location of the sound-congruent picture.

Consistent findings have been observed in the context of visual search. For instance, Knoeferle and colleagues [17] demonstrated that product jingles and product usage sounds could effectively bias visual search and eye movements in the context of online search. Iordanescu and Guzman-Martinez [15] presented participants with displays that included pictures of natural objects/animals, together with a centrally presented noninformative sound. The name of the to-be-searched-for target was presented auditorily at the beginning of each trial. Faster target localization was observed when the target object (e.g., a picture of a cat) was presented together with a semantically congruent sound (i.e., a meowing sound), as compared to when an unrelated sound or else a sound associated with a distractor picture was presented instead (see also [16]). More recently, Kvasova and colleagues [18] extended these findings in real-world scenes, designing a visual search task with dynamic scenes, such as short video clips. Consistent with the previous literature, these researchers found that semantically congruent sounds improved search performance of task-relevant visual objects embedded in the video clips. At the same time, however, object sounds failed to increase the salience of task-irrelevant distractors, as evidenced by the fact that target search performance was not slowed down by an object sound that was associated with a nontarget distractor object in the scene. Interestingly, semantically congruent associations have also been demonstrated in other sensory modalities. For instance, smelling an odor (e.g., the odor of an orange) entails faster selection and processing of odor-related visual stimuli in a scene (e.g., the picture of an orange [19,20]). Overall, the literature reveals that crossmodal semantic congruence can facilitate stimulus processing, at least under the appropriate experimental circumstances.

Crossmodal semantic congruence has also been shown to facilitate long-term episodic memory. For instance, Heikkilä and colleagues [21] investigated the impact of audiovisual encoding on later unisensory recognition memory performance. At encoding, the participants had to memorize auditory or visual stimuli (sounds, pictures, spoken words, or written words) that were presented with either a semantically congruent, incongruent, or neutral (i.e., nonsemantic noise) stimulus in the other modality. Improved subsequent memory performance was reported following the presentation of crossmodal semantic congruent stimuli, as compared to the other conditions (see also [22,23]; for a review, see [24]). Paradoxically, however, it has yet to be ascertained, at least to the best of our knowledge, as to whether crossmodal semantic congruence can affect the early stages of post-perceptual processing, such as short-term memory (STM) as well. Some evidence suggests that STM for matching crossmodal stimuli is more accurate than STM for mismatched crossmodal stimuli or else unisensory stimuli (e.g., [25,26,27,28,29]; see, for a review, [30]). The main aim of the present study was therefore to investigate whether a similar STM facilitation effect might occur following the presentation of crossmodal semantically congruent, as compared to incongruent audiovisual stimuli.

In the present study, we also assessed whether the impact of crossmodal semantic congruence on STM would be further mediated by the visual consistency of the background scene. To this end, we considered the relationship between the target object presented in the STM task and the visual scene, comparing in- vs. out-of-context visual targets while, at the same time, ruling out possible low-level confounding factors, such as any differences in the perceptual salience of the target [31]. Information that is congruent with existing schemas (such as visual scenes) is usually remembered better than less congruent information (see, e.g., [32] and for a review [33]). Other evidence, however, has demonstrated that incongruent, schema-inconsistent information is sometimes remembered better, likely because of its novelty. Accordingly, several studies have highlighted that out-of-context targets tend to capture attentional resources ([34,35,36,37,38,39,40,41,42]; though, for inconsistent findings, see [43,44]). Contextually mismatching objects are also more likely to be retrieved successfully after a short delay period [42,45,46]. It is, though, worth noting that context-incongruent facilitation appears to be extinguished at longer memory delays [47,48]. In the present study, we wanted to assess whether, and how, STM is affected by the interplay between crossmodal semantic congruence and the contextual congruence of the target object.

At encoding, the participants were presented with everyday visual scenes for a short period (500 ms). Simultaneously with the presentation of the visual scene, an object sound was also presented. This could either be semantically related to the object serving as the STM target at retrieval or not, thus defining crossmodal semantically cued vs. uncued targets. The target could also either be in- or out-of-context with respect to the scene. The capability of out-of-context targets to grab attentional resources at encoding was further manipulated across two experiments conducted on a new group of participants: in Experiment 1, each scene included two different out-of-context objects, whereas in Experiment 2, the scenes included only one out-of-context object, thus further increasing the likelihood that this single object would capture attention and hence be processed (as compared to the two out-of-context objects presented in Experiment 1). In both experiments, after a maintenance period of 2000 ms, the target was presented at retrieval in isolation against a neutral background, in the same or in a different location as the original scene. The participants had to judge the same vs. different position of the object and then provide a confidence judgment related to their previous response. If crossmodal semantic congruence were to affect STM performance, then we would expect to find increased accuracy for crossmodal semantically cued vs. uncued targets. If this effect were to be further modulated by the visual consistency of the scene, we would expect a larger cuing effect for in-context than for out-of-context targets, especially when just one out-of-context object was presented in the scene. Such a result would help to indicate that contextually incongruent objects capture attentional/encoding resources and are selected and prioritized even when unpaired with an object sound (i.e., in uncued trials).

## 2. Experiment 1

### 2.1. Methods

#### 2.1.1. Participants

Thirty healthy volunteers (16 males, mean age 22.1 ± 2.1 years) took part in the experiment. All of the participants were naive about the purpose of the experiment and reported normal or corrected-to-normal vision and normal hearing. All participants were included in the statistical analyses, i.e., none of the participants were excluded for poor performance or other reasons. The sample sizes for this and the following experiment were estimated on the basis of a power analysis conducted with G*Power 3.1.9.2 that indicated a minimum number of 24 participants for a repeated-measures analysis of variance (ANOVA), taking into account an assumed effect size of medium level (0.25), a power value of 80%, and significance level of 0.05.

#### 2.1.2. Stimuli and Materials

**Figure 1:**
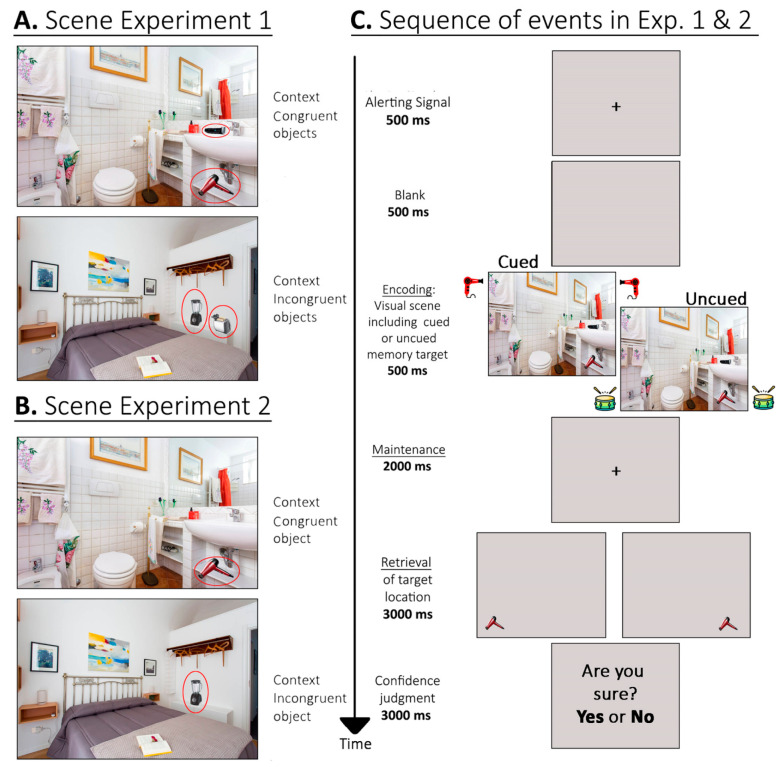
(**A**) Examples of the kinds of visual scene used in Experiment 1, involving either the inclusion of two contextually congruent objects (top panel) or two contextually incongruent objects (bottom panel). (**B**) Examples of visual scenes used in Experiment 2, involving either the inclusion of one contextually congruent object (top panel) or one contextually incongruent object (bottom panel). (**C**) Sequence of events in both experiments: each trial started with an alerting signal represented by a central fixation cross for 500 ms. After a blank screen for a further 500 ms, the visual scene was presented for 500 ms, together with an object sound that could cue an object in the scene or not. After a maintenance interval of 2000 ms, a single object was presented at retrieval, in the same or in a different spatial location, as compared to the original scene. The participants had to make a same vs. different object location judgment within 3000 ms and then provide a confidence judgment (yes/no) related to their previous response, again within a further time window of 3000 ms.

The set of stimuli included 72 different background pictures depicting everyday indoor or outdoor scenes. The scenes did not include living elements such as people or animals and were displayed at a size of 18 × 12 degrees of visual angle. Using photo editing software (Photoshop, Adobe Systems Incorporated, San Jose, CA, USA), two different objects were inserted within each scene, either on the same or opposite sides (i.e., both on the left, both on the right, or one on either side). Object size was adjusted to fit with the context of the related scene. The two objects were either both congruent or incongruent with respect to the context of the scene (see Figure 1). One of the objects served as the visual target for the STM task. Inserted objects belonged to one of eighteen different categories of common objects that are usually paired with object sounds, that is: alarm clock; blender; bongo; camera; can; cuckoo clock; electric razor; guitar; hairdryer; intercom; lighter; mobile phone; motorbike; piggy bank; vacuum cleaner; toaster; violin; washing machine. Each of the eighteen categories included four exemplars, giving rise to a total of 72 objects. Each object was inserted twice in the 72 scenes (i.e., it was presented in two different scenes), and it only served once as a target for the following STM test. Overall, half of the scenes included target objects that were contextually congruent (see, e.g., in Figure 1A, top panel, the electric razor and the hairdryer in the bathroom), while the other half included target objects that were out-of-context (see, e.g., in Figure 1A, bottom panel, the bongo and the guitar in the kitchen). In order to minimize uncontrolled effects attributable to the specific correspondence between a given scene and the inserted objects, two different lists of stimuli, A and B, each including the 72 scenes, were constructed. If a given scene in List A included congruent inserted objects, the same scene in List B included incongruent objects instead. Presentation of the lists was counterbalanced across participants. Trials within each list were fully randomized for each participant.

An object sound was presented from the two loudspeakers located on the left and right of the display at the same time as the presentation of the scene. The sounds belonged to the same eighteen categories as the inserted objects. Each object sound had a duration of 500 ms, with no gaps. Short object sounds (e.g., the lighter) could be repeated twice within the 500 ms interval. The sound volume was adjusted to be “clearly audible” for each participant, that is, until the participant was fully satisfied with the sound volume (range = 58–72 dB). In half of the scenes, the sound matched the inserted object serving as the target (e.g., the sound of a hairdryer when the hairdryer was the target object in the scene; “cued” trials; see Figure 1C); in the other half of the scenes, the sound did not correspond to the target object nor to the other inserted object or any other object in the scene (e.g., the sound of the bongos when no bongos were included in the scene; “uncued” trials; see Figure 1C). Note that all of the scenes were presented with a synchronized sound. Overall, each participant was presented with 18 scenes for each of the following target conditions: congruent cued; congruent uncued; incongruent cued; incongruent uncued.

To rule out the possibility that the congruency and cuing factors under investigation here were affected by low-level target features, we ensured that the visual saliency of the target did not vary significantly across the four experimental conditions. Target saliency was measured in each scene using the Saliency Toolbox 2.2 ([31]; http://www.saliencytoolbox.net/; accessed on 10 July 2017). This toolbox computes saliency maps based on local discontinuities in line orientation, intensity contrast, and color opponency. Next, we conducted an analysis of variance (ANOVA) on the target saliency values for the four experimental conditions. This analysis failed to reveal any significant difference [*F*(3, 140) = 1.340; *p* = 0.264; *ƞ*^2^ = 0.028], showing that target salience did not differ across conditions. This null result allowed us to rule out the possibility that performance in one condition might be favored by the inclusion of targets that, by chance, just so happened to be more perceptually salient than in the other conditions.

#### 2.1.3. Procedure

The presentation of the stimuli was conducted with Cogent 2000 (http://www.vislab.ucl.ac.uk/cogent_2000.php; accessed on 27 October 2017) running on MATLAB 7.1 (The MathWorks Inc., Natick, MA, USA). Figure 1C illustrates the sequence of events in a sample trial, starting with the presentation of a central fixation cross alerting signal, for 500 ms. After a blank screen of 500 ms, a visual scene and a sound were presented simultaneously for 500 ms (encoding phase). The sound could be associated with an object in the visual scene or not (i.e., cued vs. uncued trials, respectively; see Figure 1C). After a maintenance interval of 2000 ms, the retrieval phase began. This consisted of the presentation of a single target object (i.e., one of the two inserted objects), located in the same or in the opposite location (with respect to the central vertical meridian) occupied by the object in the encoding scene. The participants had up to 3000 ms to judge the spatial position of the target by pressing one of two response buttons with the index finger of either hand. The participants were instructed to take their time and to try to respond as accurately as possible, within the time limit. After a button press, or 3000 ms had elapsed (i.e., a missed response), the participants had to provide a confidence judgment. For this, an “Are you sure?” display was presented, requiring a “yes” or “no” response by pressing one of the two response buttons, again with a time limit of 3000 ms (otherwise missed response). The participants were instructed to provide a “yes” response if, and only if, they could vividly recollect the target object in the scene and to provide a “no” response in all the other cases (see, for a similar procedure, [45,49,50]). A new trial started after an intertrial interval of 2000 ms.

The experiment included 72 trials and lasted approximately 14 min in total. Before starting the experiment, the participants familiarized themselves with the task. For this, they were presented with a short training session that included 6 trials based on scenes, targets, and sounds not used in the main experiment.

#### 2.1.4. Data Analysis

We collected and analyzed the percentage of accuracy to recognize the spatial position of the target at retrieval (same or different, as compared to the position in the original scene) and the percentage of confidence (“Yes, I’m sure” confidence judgments) for the different experimental conditions. We also computed for each condition a sensitivity measure, the d-prime, according to the formula: d-prime = z (hits rate) − z (false alarm rate) [51]. “Hits” were defined as targets appearing at retrieval in the same position as at encoding that were correctly identified (“same” response), while “false alarms” were defined as targets located in a different position at retrieval that were incorrectly recognized as matching the original scene (“same” response). A higher d-prime indicates more accurate participants’ performance. Each measured variable (accuracy, sensitivity, and confidence) was analyzed by means of a 2 × 2 repeated-measures ANOVA with the factors crossmodal semantic cuing (target cued vs. target uncued) and target contextual congruence (target congruent vs. target incongruent). The analyses were conducted with Statistical Package for the Social Sciences (SPSS) v. 23. Individual means for each subject and measured variable are available in the online Supplementary Material (Sheet S1 & S2).

### 2.2. Results and Discussion

**Figure 2:**
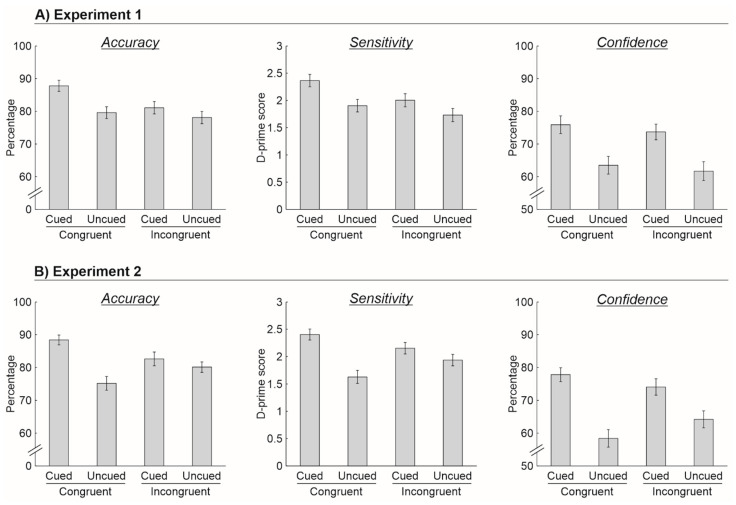
Participants’ performance in Experiment 1 (**A**) and Experiment 2 (**B**) in terms of mean accuracy, sensitivity, and response confidence as a function of crossmodal semantic cuing (cued vs. uncued targets) and target contextual congruence (congruent vs. incongruent targets). In each graph, the error bars represent the standard error of the mean.

Overall, there were only few missed responses across participants, either when responding to the spatial location task or at the related confidence judgment (0.05%), indicating that the response time interval of 3000 ms was appropriate for this task. Figure 2A illustrates the overall participants’ performance for the different experimental conditions. The ANOVA on the accuracy data revealed a significant main effect of crossmodal semantic cuing [*F*(1, 29) = 15.8, *p* < 0.001, *ƞ*^2^ = 0.353], indicating that participants’ responses were more accurate for cued (84.4%) than for uncued (78.9%) targets. The ANOVA also revealed a significant main effect of target contextual congruence [*F*(1, 29) = 11.8, *p* = 0.002, *ƞ*^2^ = 0.288], indicating greater accuracy for contextually congruent (83.7%) than for contextually incongruent targets (79.6%). However, there was no significant interaction between the two factors [*F*(1, 29) = 2.3, *p* = 0.135, *ƞ*^2^ = 0.075]. This pattern of results was confirmed by the ANOVA on the d-prime scores that revealed both the main effect of crossmodal semantic cuing [*F*(1, 29) = 16.5, *p* < 0.001, *ƞ*^2^ = 0.362] and target contextual congruence [*F*(1, 29) = 12.2, *p* = 0.002, *ƞ*^2^ = 0.295], but not the interaction effect [*F*(1, 29) = 0.9, *p* = 0.340, 1, *ƞ*^2^ = 0.031]. Finally, the ANOVA on the confidence data revealed a significant main effect of crossmodal semantic congruence [*F*(1, 29) = 45.4, *p* < 0.001, *ƞ*^2^ = 0.610], indicating more confident responses following cued (74.8%) as compared to uncued trials (62.6%), while neither the main effect of target contextual congruence [*F*(1, 29) = 1.5, *p* = 0.231, *ƞ*^2^ = 0.049] nor the interaction between the two factors [*F*(1, 29) = 0.01, *p* = 0.911, *ƞ*^2^ < 0.001] were significant.

Overall, these findings appear to reveal that both factors had an impact on STM performance. When the target object was accompanied by the relevant object sound (i.e., crossmodal semantic cued trials), STM performance was significantly better, resulting in increased accuracy, sensitivity, and confidence judgments. Analogously, STM performance (but not confidence) increased for contextually congruent targets. However, visual inspection of Figure 1A appears to tell a different story, in which the impact of object sounds (crossmodal semantic cues) is larger for contextually congruent than for incongruent targets (compare Bar 1 vs. the other bars in the accuracy and sensitivity graphs). To further test this potential interaction between the two factors on an independent sample of participants, we conducted a second experiment. Importantly, in Experiment 2, only one out-of-context object was presented within each scene, thus increasing the chance of detecting and selecting at encoding this unique, “deviant” object in terms of the overall meaning of the scene, irrespective of the crossmodal semantic cuing condition. It was expected that the uniqueness of the out-of-context object in the scene would increase the chances of this object being selected and processed at encoding, thus further reducing, or even abolishing, the effect of crossmodal semantic cuing (i.e., an interaction effect between the two factors).

## 3. Experiment 2

### 3.1. Methods

#### 3.1.1. Participants

Twenty-five healthy volunteers (15 males; mean age 24.7 ± 2.0 years) took part in the experiment. None of them took part in Experiment 1. All of the participants reported normal or corrected-to-normal visual acuity and normal hearing and were included in the statistical analyses.

#### 3.1.2. Stimuli and Materials

The set of stimuli was the same as for Experiment 1, but now we removed from each scene the out-of-context object that was not used as a target for the STM task in the previous experiment. To rule out possible differences from Experiment 1 due to the specific targets used, we kept the target the same as in the previous experiment. As in Experiment 1, two lists of stimuli, A and B were used, counterbalanced across participants, to vary the relationship between targets, scenes, and contextual congruence, i.e., each scene had a target object in List A (e.g., contextually congruent) and another target object in List B (e.g., contextually incongruent). Moreover, we once again confirmed that in these new versions of the visual scenes, the mean salience of the target objects did not differ across the four experimental conditions [*F*(3, 140) = 1.225; *p* = 0.303; *ƞ*^2^ = 0.026].

#### 3.1.3. Procedure and Data Analysis

The procedure and the approach to the data analysis were exactly the same as in Experiment 1.

### 3.2. Results and Discussion

In this experiment, there were no missed responses. Figure 2B illustrates the overall participants performance for the different experimental conditions. Once again, the ANOVA revealed a significant main effect of crossmodal semantic cuing [*F*(1, 24) = 18.4, *p* < 0.001, *ƞ*^2^ = 0.434], indicating a greater accuracy at the STM task when the participants were required to judge the spatial location of cued (85.5%) as compared to uncued (77.7%) targets. Differently from Experiment 1, the main effect of target contextual congruence was not significant [*F*(1, 24) = 0.01, *p* = 0.946, *ƞ*^2^ < 0.001], but the ANOVA revealed a significant interaction between the two factors [*F*(1, 24) = 11.7, *p* = 0.002, *ƞ*^2^ = 0.328], indicating greater accuracy for cued than for uncued contextually congruent targets (88.4% vs. 75.2%) compared to contextually incongruent targets (82.6% vs. 80.1%). These findings were fully confirmed by the ANOVA on the d-prime scores that revealed a significant main effect of crossmodal semantic cuing [*F*(1, 24) = 19.3, *p* < 0.001, *ƞ*^2^ = 0.445] and a significant interaction [*F*(1, 24) = 8.2, *p* = 0.008, *ƞ*^2^ = 0.255], while the main effect of target contextual congruence was not significant [*F*(1, 24) = 0.4, *p* = 0.552, *ƞ*^2^ = 0.015]. Finally, the ANOVA on the confidence data revealed a main effect of crossmodal semantic cuing [*F*(1, 24) = 58.1, *p* < 0.001, *ƞ*^2^ = 0.708], indicating more confidence following cued (76.1%) than uncued trials (61.3%). While the main effect of target contextual congruence was not significant [*F*(1, 24) = 0.2, *p* = 0.629, *ƞ*^2^ = 0.010], the ANOVA also revealed a trend in the interaction effect [*F*(1, 24) = 4.0, *p* = 0.057, *ƞ*^2^ = 0.143], indicating a tendency in providing more confident responses for cued vs. uncued contextually congruent targets (77.8% vs. 58.4%) compared to contextually incongruent targets (74.4% vs. 64.1%).

Overall, these findings revealed that under appropriate circumstances crossmodal semantic cuing and target contextual congruence can interact to modulate STM performance. We found that the STM retrieval of a target object presented in a visual scene was enhanced by the simultaneous presentation of a semantically congruent object sound at encoding. However, this was particularly true when the target object was contextually congruent with the visual scene. By contrast, the STM retrieval of contextually incongruent targets was much less affected by the object sound, especially when only one incongruent target object was included in the scene as in Experiment 2; See the General Discussion (Section 4) on this point.

## 4. General Discussion

The main aim of the present study was to assess whether crossmodal semantic congruence modulates performance at the post-perceptual level, as assessed in an STM task, and whether this effect was further modulated by the target contextual congruence with the visual scene. The participants had to judge the spatial position of a target object that had previously been presented within an everyday scene. The target object in the scene could either be presented with the related object sound or not. Moreover, the target object could be in- or out-of-context, as compared with the visual scene. Across two experiments, in which the number of out-of-context objects in the scene was manipulated systematically, a consistent effect of crossmodal semantic congruency was observed. Specifically, in both experiments, greater accuracy was observed for those targets that were cued by semantically coherent sounds at encoding (i.e., during scene presentation) as compared to uncued targets. This crossmodal facilitatory effect decreased when the STM target object was out-of-context, in those scenes including only one out-of-context object (i.e., in Experiment 2).

The impact of crossmodal semantic congruence on the spatial allocation of attentional resources has already been demonstrated by previous studies within the context of visual search tasks. This literature has revealed that objects paired with semantically congruent sounds are located more rapidly within displays of isolated objects [15,16,17,52] or within dynamic scenes [18]. This effect was generally interpreted as a consequence of the increased salience derived from the congruent crossmodal signals that, in turn, promoted stimulus prioritization over the other stimuli [53,54,55,56,57]. The existence of a semantic-related salience (as compared to a perceptual-related salience; see [58] on this point) is in good agreement with the notion that visual attention is strongly guided by semantic information and previous knowledge in real-world contexts ([59,60,61,62]; for reviews, see [63,64,65,66,67,68]). Here we extended this finding at a post-perceptual level, demonstrating that increased semantic-related salience can affect encoding prioritization, thus resulting in greater STM performance at retrieval. 

Several studies and theories have proposed that the probability of successfully retrieving STM information is a function of attentional prioritization at the encoding stage (e.g., [69,70]; see, for reviews, [71,72,73,74,75]). Here we extended this notion by showing that STM performance is enhanced as a consequence of crossmodal semantic congruence at encoding. This effect was interpreted as a facilitation in selecting and processing visual stimuli paired with semantically coherent sounds at encoding, with a subsequent increase in the chance to remember the spatial position of the object later at the retrieval stage. The integration of audiovisual semantically coherent information may take place automatically, or rather be dependent on top-down processes, related to the observer’s assumption that the two unisensory components belong to the same object (i.e., the so-called “unity assumption” [76]). If the integration of audiovisual semantic congruent information is affected by top-down processes, this effect may well involve post-encoding stages, such as the maintenance period. For example, according to Chen and Spence [13,14], congruent sounds may influence both the earlier and later stages of visually-related information processing (though see [77]). When the auditory input is “salient” enough to be selected and processed (e.g., [78]), its meaning may unavoidably interact with any relevant visual information, in agreement with the Conceptual Short-Term Memory model first proposed by Potter [79,80]. According to this model, the meaning of a picture is accessed rapidly (within the first 100–200 ms), and the activated semantic representation is then retained for a further 300–500 ms within Conceptual Short-Term Memory (see also [81]). The semantic representation of the picture, as well as other relevant semantic information, is then available for a certain amount of time that can extend from the encoding to the maintenance stage to be integrated into a unique percept (see [82,83] for reviews). Influences at both earlier and later stages of information processing may therefore account for the effect of crossmodal semantic congruence on STM within the current experimental setting.

The current experimental design allowed us to move a step forward and highlight that the crossmodal facilitatory effect of object sound on STM performance also depends on the specific semantic configuration of the visual scene where the target object is embedded, that is, on whether the target object was congruent or not with the context of the visual scene. This was particularly true when only one context-incongruent object was included in the to-be-encoded visual scenes (Experiment 2), likely increasing the chance of selecting and processing this stimulus (see, e.g., [34,35,37,39,40,41]). This effect is interesting in the light of the previous literature on this topic, which reported mixed results. For example, Ortiz-Tudela and colleagues [47] reported that context-incongruent objects were detected more rapidly than context congruent objects, but they resulted in more discrimination errors in a recognition task. Ortiz-Tudela et al. [48] also reported that incongruent objects were better detected but worse identified. Interestingly, while the previous studies that documented effects related to out-of-context targets typically used a much longer stimulus exposure duration, in the current experiments, we used a short presentation of the visual scenes (i.e., 500 ms). For instance, the visual scene was displayed until participants responded in Underwood and Foulsham’s [42] study (Experiment 1), while in Silva and colleagues’ [46] study, the scenes were displayed for 15 s; in Santangelo and colleagues’ [45] study, the scenes were presented for 4 s. In Ortiz-Tudela and colleagues’ [47] study, the participants required an average of approximately 2600 ms in order to find contextually incongruent targets. Spotorno and colleagues [40] and Stirk and Underwood [41] both presented visual scenes for shorter durations (120 ms and 480 ms, respectively), but the presentation of each scene was repeated more times after short delays, in a change detection paradigm. Here we used a delayed match-to-sample task [84] with a short encoding time of 500 ms in order to tap into the STM memory representation of visual scenes. Using such a brief presentation interval, we documented an effect of out-of-context targets embedded in complex scenes rich in competing objects. In particular, we showed an interplay with crossmodal semantic cuing, especially when only one contextually incongruent target was included in the visual scene (i.e., Experiment 2). The out-of-context target dramatically reduced the impact of crossmodal semantic cuing. STM retrieval of contextually congruent visual targets was better when the visual target was paired with sound objects (as compared to when it was not paired with the object sound, i.e., cued vs. uncued trials). By contrast, STM retrieval of out-of-context targets was similar irrespective of whether the visual target was paired or not with the object’s sound. Incidentally, in Experiment 2, the difference in the percentage of confident responses for cued vs. uncued trails also tended to be reduced when the visual target was contextually incongruent. Overall, these findings highlight a clear impact of prior experiences on STM retrieval (see, e.g., [85]) in terms of audiovisual semantic-related processing, suggesting that both crossmodal and scene-related semantic congruence interact during the encoding and subsequent SMT retrieval of complex visual scenes. We suggest that selection at encoding is strongly driven by object sounds for in-context visual objects, while this effect is dramatically reduced for out-of-context objects that are prioritized and selected even when unpaired with the object sound.

Finally, a common limitation to those studies involving complex, real-world stimuli, such as real-world scenes or videos, is worth mentioning. This stimulus material is highly heterogeneous and can be categorized using a virtually unlimited number of parameters. For instance, a clear impact on attention-related mechanisms at encoding is played by stimulus complexity, that is, the number of objects that happen to be competing for attention (and hence perceptual processing). This is, however, not a trivial issue to resolve, as the number of objects within a given scene strictly relies on one’s definition of an object: for instance, if a scene includes a car, should the car be counted as a single object, or should each single part of the car be counted as an object? The general global configuration of the scene has also been shown to affect participants’ performance [63,64]. Here, we tried to compensate for the heterogeneity in stimulus complexity/configuration by building two different stimulus lists, counterbalanced across participants, in which we varied the inclusion of a given target in a given scene. We also controlled low-level sensory features of the target objects, such as the perceptual salience, that have been shown by previous research to modulate perceptual and post-perceptual processes (see [58] for a review). Notwithstanding that, we acknowledge that uncontrolled factors can potentially contribute to findings reported using real-world material.

## 5. Conclusions

To conclude, enhanced STM performance was demonstrated for in-context visual targets embedded in complex and realistic scenes that happened to be cued by semantically congruent sounds, while this crossmodal semantic facilitatory effect dramatically decreased for out-of-context visual targets. Within the current experimental settings, the object sound therefore appeared efficient in promoting the STM representation, and the related retrieval, of the target than the object contextual congruence, but this also depended on the semantic configuration of the visual scene, with out-of-context targets being selected and prioritized even when unpaired with the object sound. Overall, these findings revealed that crossmodal semantic congruence interacts with semantic configuration of the visual scene and the related object consistency to modulate post-perceptual performance, at the level of STM.

## Data Availability

The data presented in this study are available in the Supplementary Material.

## Supplementary Materials

The following are available online at https://www.mdpi.com/article/10.3390/brainsci11091206/s1, Sheet S1: Data Experiment 1, Sheet S2: Data Experiment 2.
